# Implementation of aspirin use during pregnancy in community midwifery-led care in the Netherlands: A pilot survey

**DOI:** 10.18332/ejm/191161

**Published:** 2024-08-01

**Authors:** Jeske M. bij de Weg, Rebecca van Doornik, Kim L.H.E. van den Auweele, Christianne J.M. de Groot, Marjon A. de Boer, Johanna I.P. de Vries

**Affiliations:** 1 Department of Obstetrics and Gynaecology, Amsterdam University Medical Center, Amsterdam, the Netherlands; 2 Royal Dutch Association of Midwives, Utrecht, the Netherlands; 3 Dutch HELLP Foundation, Zwolle, the Netherlands

**Keywords:** implementation, pregnancy, aspirin, hypertensive disorders of pregnancy, community midwives

## Abstract

**INTRODUCTION:**

Aspirin nowadays is widely used in pregnancy, but implementation among gynecologists took nearly four decades. For a complete insight in the implementation of aspirin, community midwives are to be involved. Community midwives do not have authority to prescribe aspirin and have to refer to a general practitioner or consultant obstetrician for a prescription.

**METHODS:**

The study was an online, national pilot survey about the implementation of aspirin use during pregnancy among independently practicing community midwives consisting of 29 items with five categories: background, advising, prescribing, possible indications, and clinical practice.

**RESULTS:**

Forty-seven community midwives completed the survey between April and May 2021. All respondents had experience on advising aspirin use in pregnancy. History of preterm pre-eclampsia or HELLP syndrome was identified as a risk factor for developing utero-placental complications by 97.9% of the community midwives. Moderate risk factors in women with otherwise low-risk pregnancy were identified by >75% of the participants. Practical issues in prescribing aspirin were experienced by one-third of the respondents. Suggestions were made to obtain authority for community midwives to prescribe aspirin and improve collaboration with consultant obstetricians and general practitioners.

**CONCLUSIONS:**

Community midwives seem to be adequate in identifying risk factors for developing utero-placental complications in women with otherwise low-risk pregnancy. Practical issues for prescribing aspirin occur often. Obtaining authority for community midwives to prescribe aspirin after education should be considered and consulting a consultant obstetrician should become more accessible to overcome the practical issues. Further educating community midwives and general practitioners might improve implementation rates and perinatal outcomes.

## INTRODUCTION

Utero-placental insufficiency can lead to several obstetric complications, including hypertensive disorders of pregnancy (HDP) and fetal growth restriction (FGR). Utero-placental complications contribute significantly to maternal and neonatal morbidity and mortality in the short- and long-term^1-5^. Unfortunately, no curative options besides delivery exist. Therefore, preventive strategies are of great importance. Low-dose aspirin is proven to reduce the risk of HDP and FGR in women at risk^6-8^. The risk-reducing effect of aspirin on preterm preeclampsia (PE) by up to 62% has been reported^9^.

Nowadays, aspirin is widely used in pregnancy, but its risk-reducing effect has been studied for many years, initially with conflicting results^10^. The risk-reducing effect of aspirin on HDP and FGR was first demonstrated in 1985^11^. The evidence that administration of aspirin in early pregnancy before 16 weeks of gestation is crucial for its risk-reducing effect, was a break-through in 2010^12^. This insight resulted in publication of international guidelines about aspirin use during pregnancy^13-15^. Aspirin was added to the Dutch national guideline of obstetricians and gynecologists in 2018^16^. Recently, bij de Weg et al.^17^ revealed improved implementation of aspirin use during pregnancy among Dutch consultant obstetricians after publication of the national guideline between 2016 and 2021. Altogether, it took nearly four decades from first evidence about aspirin use during pregnancy to confirmed implementation of aspirin use during pregnancy among consultant obstetricians.

For a complete insight in the implementation of aspirin, community midwives are to be involved. Dutch maternity care system is based on risk selection, and >90% of newly pregnant women have their initial appointment with a community midwife. Community midwives are independently practicing healthcare providers with responsibility for the care of low-risk pregnant women. Hence, these midwives are able to identify risk factors in early pregnancy. Since timely onset of aspirin administration early in pregnancy has the optimal effect in reducing adverse outcomes, it is important that community midwives have knowledge of aspirin and its effects. Current guidelines acknowledge multiple moderate risk factors for developing utero-placental insufficiency which may not necessarily require an input from a consultant obstetrician. So, women with an otherwise low-risk pregnancy, might still have an indication for aspirin in pregnancy^13,15,16,18,19^. Examples are nulliparous with a family history of PE, women with obesity (body mass index >35 kg/m^2^) and a pregnancy interval >10 years, or women aged ≥40 years and pregnant after egg cell donation. The Royal Dutch Association of Midwives aimed to improve awareness of the benefit of aspirin use in pregnancy among community midwives through implementation of a module on their website concerning scientific summary about aspirin to reduce the risk of HDP^20^. In addition, a Dutch information folder about aspirin use during pregnancy for pregnant women under community midwives’ care was created^21^. Dutch community midwives are competent to set the indication for aspirin use in pregnancy, but do not have the authority to make a prescription for aspirin. Therefore, referral to a general practitioner or consultant obstetrician who do have the authority to prescribe aspirin is necessary.

A survey about barriers and facilitators faced by midwives in the United Kingdom showed that conflicting views and deficits in resources hindered the implementation of aspirin^22^. A systematic review on counselling about aspirin revealed three categories predicting the success of implementation of aspirin: capability, motivation and opportunity^23^.

In the present study, we investigated the implementation of aspirin in pregnancy in the community midwifery-led care in the Netherlands and its practical needs and issues.

## METHODS

### Study design and setting

This study was conducted in April and May 2021. The survey was designed by the investigators (JbdW, MdB, JdV) and a clinical midwife (RvD) of our tertiary center who is also a scientific advisor to the Royal Dutch Association of Midwives. The patients’ organization Dutch HELLP Foundation collaborated as well. The Medical Research Ethics Committee gave approval (no 2020.0651). Sample size calculation resulted in 330 participants, based on approximately 2500 registered community midwives at the time of study design, for a 5% margin of error with 95% confidence interval.

### Participants and measures

The survey was addressed anonymously to members of the Royal Dutch Association of Midwives (Koninklijke Nederlandse Organisatie van Verloskundigen, KNOV) in their weekly newsletter. Individuals who wished to participate could respond through a link provided in the newsletter. The survey could only be started after participants gave digitally informed consent. After eight weeks, a reminder was sent in a later newsletter. The survey was also available on their website^24^. In close collaboration with the association, this method was considered more anonymous and preferred over a mass mail to all midwives. Midwives in training, clinical midwives and non-practicing community midwives were excluded from participation, since we were interested in the current status of the implementation of aspirin in the community midwifery led-care.

The survey was conducted in Dutch and consisted of 29 items – multiple choice and open questions – divided into five categories: 1) characteristics; 2) advising about aspirin use during pregnancy; 3) prescribing process for aspirin administration; 4) knowledge of midwives on possible indications for aspirin use during pregnancy; and 5) clinical practice. The instrument used in the present study, based on bij de Weg et al.^17^ about the implementation of aspirin among consultant obstetricians, is listed in Supplementary file Table 1. The survey was structured in such a way that only fully completed questionnaires could be submitted. An online, anonymous survey was chosen to lower social desirability bias. Distribution of the survey was performed by LimeSurvey to ensure data protection, acknowledged by the privacy officer.

### Statistical analysis

Qualitative and quantitative variables are presented with descriptive statistics. Means with standard deviation (SD) are reported for normally distributed continuous variables, and medians with interquartile range (IQR) are reported for non-normally distributed continuous variables. Frequencies and percentages are reported for categorical variables. Statistical analysis was performed in SPSS version 27.0.

## RESULTS

Regarding participants characteristics, the survey was completed by 47 community midwives. The estimated response rate was 1.8%, 47 out of approximately 2568 registered community midwife members of the Royal Dutch Association of Midwives in 2021. General characteristics of the participants are listed in Table 1.

**Table 1:** Characteristics of community midwives participating in a survey pilot study on aspirin use during pregnancy, the Netherlands, 2021 (N=47)

*Characteristics*	*Mean ± SD*	*Median (IQR)*	*n (%)*
**Age** (years)	41.7 ± 11.9		
**Female gender**			47 (100)
**Year of graduation**		2008 (1996–2015)	
**Country of graduation**			
The Netherlands			42 (89.4)
Belgium			5 (10.6)
Other			0 (0)
**Work experience** (years)	16.0 ± 11.8		
**Clinical work experience**			
Yes			2 (4.3)
Years of experience		3.5 (1.0–3.5)	
Provinces*			
Noord-Holland			20 (40.6)
Noord-Brabant			7 (14.9)
Gelderland			5 (10.6)
Groningen			5 (10.6)
Limburg			5 (10.6)
Zuid-Holland			4 (8.5)
Drenthe			2 (4.3)
Overijssel			1 (2.1)
Friesland			1 (2.1)
Zeeland			1 (2.1)
Flevoland			0 (0)
Utrecht			0 (0)
**City, village or both**			
City			23 (48.9)
Village			17 (36.2)
Both			7 (36.2)
**Colleagues in practice**	4.6 ± 2.3		
**Pregnant women in practice**	285.8 ± 150.4		

* Can be >100% because some participants are working in multiple provinces. IQR: interquartile range.

Concerning the knowledge about aspirin use during pregnancy, 32 out of 47 (68.1%) midwives were familiar with the module about aspirin of the Royal Dutch Association of Midwives, and 14 (29.8%) midwives were familiar with the client folder. The details about advising about aspirin use during pregnancy are given in Table 2.

**Table 2:** Counselling on aspirin use during pregnancy by community midwives, a pilot survey, the Netherlands, 2021 (N=47)

*Information (Royal Dutch Association of Midwives)*	*n (%)*
**Module about aspirin use during pregnancy**	
Knowledge of module	32 (68.1)
**Client folder about aspirin use during pregnancy**	
Knowledge of client folder	14 (29.8)
If yes, hand out to clients (n=14)	
Always	4 (28.6)
Frequently	3 (21.4)
Regularly	1 (7.1)
Sometimes	0 (0.0)
Never	6 (42.9)

About prescribing aspirin, all participants had advised aspirin use during pregnancy at least once. Practical issues with obtaining prescriptions for aspirin were reported by 17 participants (36.2%). The majority of this group, 14 out of 17 participants, specified that general practitioners are unfamiliar with the effects of this drug in pregnancy and reluctant to sign prescriptions. Two out of 17 participants experienced logistic problems and costs to receive a prescription of the consultant obstetrician, and one stated that policies differ per hospital and therefore cause confusion.

A later start of aspirin usage than advised in the module (before 16 weeks of gestation) due to practical issues was mentioned by 11 out of 17 participants experiencing these practical issues. The delayed start of aspirin usage varied from 2% to 50% of the cases. Two participants reported that due to practical issues they sometimes ignore the indication for aspirin and did not provide referrals for prescriptions anymore: one in estimated 3%, and one in estimated 20% of the cases.

Various routes for prescribing aspirin were used (Table 3). The majority of the participants referred to the general practitioner for a prescription (n=19; 40.4%), followed by consultation with a consultant obstetrician (n=17; 36.2%), over the counter (‘children’s aspirin’; n=14; 29.8%) and by an obstetrician within multidisciplinary setting (without a consultation) (n=11; 23.4%). Five participants reported another route: consultation of the midwife with a consultant obstetrician by phone (no consultation of patient in hospital; n=3) or via internet (n=1), and over the counter (n=1). Around one-third preferred to get a prescription via the general practitioner, and one-quarter over the counter. Two participants preferred to have the authority to prescribe aspirin themselves.

**Table 3:** Counselling and prescribing process of aspirin administration by community midwives, a pilot survey, the Netherlands, 2021 (N=47)

	*n (%)*
**Advising aspirin**	47 (100)
**Practical issues prescribing aspirin**	17 (36.2)
Aspirin prescription options used*	
Consultation with a consultant obstetrician	17 (36.2)
Via obstetrician within obstetric multidisciplinary setting	11 (23.4)
Via general practitioner	19 (40.4)
Over the counter	14 (29.8)
Other	5 (10.6)
**Preferred options of aspirin prescription**	
Consultation with a consultant obstetrician	2 (4.3)
Via obstetrician within obstetric multidisciplinary setting	11 (23.4)
Via general practitioner	16 (34.0)
Over the counter	12 (25.5)
Other	6 (12.8)
**Instructions for the timing of aspirin administration**	28 (59.6)
**Initiation of aspirin use**	
8–12 weeks of gestation	14 (29.8)
12–16 weeks of gestation	32 (68.1)
Other	1 (2.1)
**Stop aspirin use in gestational age in weeks**	
34	0 (0.0)
36	46 (97.9)
38	0 (0.0)
Other	1 (2.1)

* Can be >100% because participants used multiple routes in daily practice.

Regarding possible indications for aspirin use during pregnancy, participants’ knowledge about indications for aspirin use during pregnancy of community midwives is listed in Table 4. History of preterm pre-eclampsia or HELLP syndrome was identified as a risk factor for developing utero-placental complications by 97.9% of the community midwives. Moderate risk factors in women with otherwise low-risk pregnancy were identified by >75% of the respondents.

**Table 4:** Possible indications for aspirin use during pregnancy, a pilot survey, the Netherlands, 2021 (N=47)

*Indications*	*Yes, as isolated risk factor n (%)*	*Yes, in combination with other risk factors n (%)*	*No, no indication n (%)*	*No, not sufficiently informed n (%)*
**Hypertensive disorders**				
PIH with at term birth	**17 (36.2)**	20 (42.6)	9 (19.1)	1 (2.1)
PE/HELLP with at term birth	**44 (93.6)**	3 (6.4)	0 (0.0)	0 (0.0)
PE/HELLP with at late preterm birth between 34 to 37 weeks of gestation	**44 (93.6)**	2 (4.3)	0 (0.0)	1 (2.1)
PE/HELLP with early preterm birth <34 weeks of gestation	**46 (97.9)**	0 (0.0)	1 (2.1)	0 (0.0)
**Fetal growth restriction**				
FGR with at term birth	**28 (59.6)**	17 (36.2)	1 (2.1)	1 (2.1)
FGR with at late preterm birth between 34 to 37 weeks of gestation	**31 (66.0)**	10 (21.3)	1 (2.1)	5 (10.6)
FGR with early preterm birth <34 weeks of gestation	**32 (68.1)**	9 (19.1)	1 (2.1)	5 (10.6)
**Auto-immune diseases**				
SLE	**21 (44.7)**	0 (0.0)	1 (2.1)	25 (53.2)
APS	**22 (46.8)**	1 (2.1)	0 (0.0)	24 (51.1)
**Maternal illness**				
Pre-existent hypertension	**36 (76.6)**	5 (10.6)	1 (2.1)	5 (10.6)
Diabetes type I or II	**24 (51.1)**	3 (6.4)	7 (14.9)	13 (27.7)
Kidney diseases	**25 (53.2)**	2 (4.3)	5 (10.6)	15 (31.9)
**Moderate risk factors**				
Nulliparity	0 (0.0)	**39 (83.0)**	8 (17.0)	0 (0.0)
Advanced maternal age ≥40 years	5 (10.6)	**41 (87.2)**	0 (2.1)	0 (0.0)
Pregnancy interval >10 years	0 (2.1)	**36 (76.6)**	6 (12.8)	4 (8.5)
BMI >35 kg/m^2^	5 (10.6)	**38 (80.9)**	37 (4.3)	1 (4.3)
Positive family history of HDP	4 (8.5)	**6 (78.7)**	4 (8.5)	2 (4.3)
Multifetal pregnancy	8 (17.0)	**25 (53.2)**	38 (8.5)	10 (21.3)
Egg cell donation	6 (12.8)	**28 (59.6)**	4 (8.5)	9 (19.1)
Perinatal death due to placental insufficiency	31 (66.0)	**12 (25.5)**	0 (0.0)	4 (8.5)
Unexplained perinatal death	21 (44.7)	**16 (34.0)**	2 (4.3)	8 (17.0)

PIH: pregnancy induced hypertension. PE: pre-eclampsia. HELLP: hemolysis elevated liver enzymes low platelets. FGR: fetal growth restriction. SLE: systemic lupus erythematosus. APS: anti-phospholipid syndrome. HDP: hypertensive disorders of pregnancy. The correct answers according to the Dutch national guideline and module of the Royal Dutch Association of Midwives, based on ACOG and NICE guidelines, are depicted in bold^13,14,16,20^.

Regarding the preferred source of obtaining information on aspirin use during pregnancy, e-learnings were selected most frequently (n=24), followed by extending the module about aspirin of the Royal Dutch Association of Midwives (n=5), providing a national website about the topic (n=5), regional contact person (n=1), and written information of other resources (n=1). Eight participants selected the option ‘Other’, six suggested to study the module more in detail, one suggested a regional folder, and one mentioned that aspirin indications are medical indications.

The great majority of the midwives (n=45; 95.7%) reported that the intake consultation of the pregnant women is sufficiently early in pregnancy for timely start of aspirin before 16 weeks and of ample duration to sufficiently inform pregnant women (n=42; 89.3%). Around two-thirds of the participants (n=30; 63.8%) checked the adherence of aspirin regularly or frequently and used the website of The Netherlands Pharmacovigilance Centre Lareb^25^ as a source to explain safety of the drug in pregnancy (n=33; 70.2%). The majority of the participants (n=27; 57.8%) almost never checked side effects.

## DISCUSSION

The main finding of this study is that community midwives considerable contribute to the implementation of aspirin use during pregnancy, since all participants advised aspirin in their practice. Moreover, the majority knew their professional association’s website module on aspirin advise, and demonstrated their knowledge by the considerable rates of identifying risk factors for utero-placental complications and indication for aspirin use during pregnancy. Practical issues for the community midwives concerning prescribing aspirin were mentioned by one-third of the participants.

A small proportion of the practicing community midwives in the Netherlands responded to our survey. Characteristics of the participants correspond with the overall population: mostly female gender, educated in the Netherlands or Belgium, and with a wide range on years of work experience. Most participants worked in the province Noord-Holland and in a city.

Two-thirds of the participants are familiar with the module about aspirin use during pregnancy of the Royal Dutch Association of Midwives. Early initiation of aspirin before 16 weeks of gestation reduces the risk of developing utero-placental complications^12^. Therefore, adequate identification of risk factors by the healthcare professional where the majority of pregnant women start their check-ups is of great importance. The minority of the participants was familiar with the client folder, and about half of them handed the folder out to the pregnant women sometimes. bij de Weg et al.^26^ previously investigated patients’ perspective on aspirin use during pregnancy, showing that the majority of women at risk preferred to be informed, both verbally and written. In 2020, Karunia et al.^27^ showed that offering verbal and written education about pre-eclampsia and aspirin resulted in improved knowledge and medication adherence. Evidence on compliance rate and the risk-reducing effect of aspirin showed a trend to lower incidence of preterm pre-eclampsia with an aspirin adherence ≥90% in comparison to <90% (OR=0.24 vs OR=0.65, p=0.19)^28^. Reducing adverse obstetric outcomes could be achieved by raising adherence rates through improving pregnant women’s knowledge by counselling with the client folder. Another factor to take into account is the influence of the media communicating about results of healthcare studies^29^. Press releases might influence the decision making of pregnant women. Finally, pregnant women with low health literacy should receive additional support in participating in the shared-decision making process^30^. Designing folders with pictograms could be helpful for this population.

Around one-third of the participants experienced practical issues with obtaining prescriptions for aspirin. The majority of this group described issues with receiving a prescription from general practitioners, mostly because of unfamiliarity of the general practitioner with this subject since it is not mentioned in the Dutch guidelines of general practitioners. Participants prefer to obtain a prescription through the general practitioner over the consultant obstetrician for practical reasons: more easily accessible; women can mostly just collect the prescription in contrast to having to visit the consultant obstetrician. Practical issues were previously identified in a national survey among midwives in the United Kingdom as barriers in implementation of aspirin in clinical practice^23^.

Almost 60% of the participants instructed women to take their aspirin at nighttime. Participants who did not specify these instructions, responded that this is the responsibility of the prescribing doctor or dispensing pharmacist, or that they were unfamiliar with this advice. The client folder informs about preferred time, but does not describe the rationale behind it. Platelets have a circadian rhythm with a peak release in the late night and early morning^31^. To inhibit this peak, nighttime intake helps rapid absorption with time to maximal plasma levels of up to two hours. Thereby, a more stable platelet inhibition >24 hours was seen in the cardiovascular population in case of nighttime intake in comparison with morning intake^32^. Education about this topic might motivate an increasing number of community midwives to optimize instructions.

There is consensus about the advice to start aspirin before sixteen weeks of gestation and advocated by the Royal Dutch Association of Midwives, the national guideline of the Dutch Society of Obstetrics and Gynecology and the international guidelines^13,15,16,18-20^. The beneficial effect of an early start of aspirin intake was reported by Bujold et al.^12^. Aspirins risk-reducing effect is based on improvement of placentation, a process that takes place during the first and early second trimester^33^. Also mentioned in guidelines, is the consensus to stop aspirin at 36 weeks of gestation^13,15,16,18-20^. There is no scientific evidence for this advice; however, there seems to be a biologically plausible reason, based on the life span of platelets between seven to ten days and the term period of pregnancy starting from 37 weeks of gestation onwards. Low-dose aspirin usage during pregnancy might slightly increase the risk of postpartum hemorrhage, but evidence is of moderate quality^6,34^. Timely discontinuation may decrease the risk of bleeding around labor and promote guidance of community midwives during labor in low-risk women. The fact that there is no contraindication for regional analgesia during the use of aspirin without other anticlotting medication, confirms the safety profile of aspirin^35^.

The present study listed several possible indications for aspirin use during pregnancy; the highest identification rates were seen for the indications history of PE and HELLP syndrome. Lower rates were found for history of term pregnancy induced hypertension (PIH) and for FGR. Furthermore, this might be explained by the scientific evidence showing a stronger risk-reducing effect of aspirin on PE in comparison to term PIH and to FGR^7^. On the other hand, placenta-related complications occur more often at term and therefore justify the counselling about aspirin for consecutive pregnancies^13,18^.

The present study revealed that community midwives will most frequently identify an indication for aspirin in case of a combination of two or more moderate risk factors. Reasonable identification rates (>75%) were found for combinations of nulliparity, advanced maternal age (≥40 years), long pregnancy interval (>10 years), obesity and a family history of PE. In 2016, Bartsch et al.^36^ developed a list of clinical risk factors for caregivers to identify women at risk for PE. In their meta-analysis, these factors were also identified as risk factors for PE^36^.

Half of the participants preferred e-learning to improve their knowledge about aspirin use during pregnancy. A Cochrane review compared the effect of e-learning with traditional learning methods in a mixed professional care givers population, showing a similar or slightly better effect of e-learning on long-term skills and knowledge and in patients outcomes^37^.

Participants felt no need for an earlier or longer intake consultation to improve counselling about aspirin use during pregnancy. More than half of the participants checked for adherence. The growing caregivers’ awareness of the effect of aspirin adherence on clinical outcomes as demonstrated among gynaecologists^17^ might increase the number of caregivers addressing this topic. Vigilance among midwives concerning knowledge on and checking for side effects should be standardized, because experiencing complaints or side effects could negatively influence adherence.

### Strengths and limitations

Investigating implementation of clinical guidelines among all type of caregivers should be performed to facilitate improvement and we contributed with evaluations among community midwives in the Dutch setting. Besides evaluating implementation rates, these midwives had the opportunity to explain practical issues they were confronted with. This study was a unique collaboration between consultant obstetricians, a midwife, and a patient organization on this subject. The most important limitation of our study is the low response rate, this hindered the original set-up and the data resulted in a modest pilot study. The low response is probably based on the method of distribution of our survey. A mass mail specifically about the survey may have led to a higher response. The low response could be an indication of unfamiliarity and/or disinterest in the subject. On the other hand, this is in contrast with the good implementation of aspirin among the participants. The results of our pilot study can function as a base for further research.

### Implications for practice

Community midwives seem to facilitate the implementation of aspirin in women at risk for developing utero-placental complications. Attention on and dissemination of the module about aspirin of the Royal Dutch Association of Midwives could help to expand knowledge and implementation. Issues to improve are the instructions to take aspirin at nighttime and checking for adherence. Education in the form of e-learning was preferred in half of the participants. To overcome practical issues with prescribing aspirin, several options were covered. One option was to obtain legal authority for community midwives to prescribe aspirin. We argue for the facts that: 1) low-dose aspirin is safe for mother and fetus with low incidence of side effects; and 2) its availability over the counter, justifies an extension of legal authority of midwives to prescribe aspirin, provided they received education on the topic. Alternatives are improved accessibility of consultant obstetricians for phone or digital consultations, education of general practitioners about aspirin use during pregnancy, and development of shared guidelines with general practitioners.

In addition, we would like to advice the Royal Dutch Association of Midwives to create a national guideline about aspirin use during pregnancy to help community midwives with the implementation. bij de Weg et al.^17^ showed that after the creation and publication of the national guideline of the Dutch Society of Obstetrics and Gynecology, the implementation of aspirin among Dutch gynecologists significantly improved.

## CONCLUSIONS

The present pilot study shows that the participating community midwives adequately identified risk factors for developing utero-placental complications in women with otherwise low-risk pregnancy. This knowledge attributes to the significance of community midwives in contributing to the implementation of aspirin use during pregnancy. The finding that the most common practical issue was prescribing aspirin, should be evaluated whether authorization of community midwives can be facilitated in the near future. With this study, we extended the research of implementation of aspirin use during pregnancy among caregivers. The results of our pilot study could be used for further studies to improve implementation, in collaboration with pregnant women, patient organizations, midwives, general practitioners and consultant obstetricians.

## Supplementary Material



## Data Availability

The data supporting this research are available from the authors on reasonable request.
