# Supplementary material for: Implementation of aspirin use during pregnancy in community midwifery-led care in the Netherlands: A pilot survey
Source: Eur J Midwifery. 2024 Aug 1;8:10.18332/ejm/191161. doi: 10.18332/ejm/191161 (PMC11292730; doi:10.18332/ejm/191161)
Supplement: Supplementary file 1 [file EJM-8-41-s1.pdf]

Supplementary material: information in order to obtain informed consent for survey in Dutch and English

Original information in Dutch

Introductie

Wij willen u graag uitnodigen voor deelname aan ons vragenlijstonderzoek. Hieronder volgt eerst uitleg over het nut van de vragenlijst. Met uw inzet hopen we meer te weten te komen over de implementatie aspirine tijdens de zwangerschap in de eerstelijns verloskundigenpraktijk. Het medicijn is te krijgen via de drogist of apotheek en heeft als merknaam aspirine (generieke naam acetylsalicylzuur) of ascal (generieke naam carbasalaatcalcium) en wordt tijdens de zwangerschap gegeven ter verlaging van de kans op placentaire complicaties zoals hypertensieve aandoeningen in de zwangerschap en foetale groeirestrictie bij vrouwen met een verhoogd risico hierop.

Steeds meer zwangere vrouwen hebben volgens de huidige richtlijnen een indicatie voor aspirinegebruik in de zwangerschap. Actuele kennis over placentaire complicaties in de zwangerschap en het risicoverlagende effect van aspirine is vastgelegd in de richtlijn 'Hypertensieve aandoeningen' van de NVOG sinds december 2018 en in de module 'Acetylsalicylzuur' welke gepubliceerd is in oktober 2019. De KNOV heeft in januari 2019 een module 'Ascal bij verhoogd risico hypertensie' gepubliceerd en in augustus 2020 een wetenschappelijke reactie 'Ascal bij dysmaturiteit in de anamnese'. Deze documenten worden ondersteund door een KNOV cliëntenfolder 'Aspirine'. Deze folder bevat informatie voor zwangere vrouwen met een indicatie voor aspirinegebruik in de zwangerschap. De informatie bevat onder andere de reden voor aspirinegebruik in de zwangerschap en de risicoverlaging, maar ook praktische informatie over onder andere tijdstip van inname.

Het doel van dit onderzoek is om meer inzicht te verkrijgen in:

- Verschillende aspecten rondom het adviseren van gebruik van aspirine in de zwangerschap.

- Suggesties voor verdere implementatie van aspirinegebruik bij zwangere vrouwen met indicatie.

We hebben berekend hoeveel personen de vragenlijst moeten invullen om tot een betrouwbare uitspraak te komen. Dit zijn er circa 330. Dit is gebaseerd op circa 2500 praktiserende eerstelijnsverloskundigen. Hiermee bereiken we het gewenste betrouwbaarheidsniveau van 95% en is er rekening gehouden met 5% als acceptabele foutenmarge. U snapt dat wij uw inzet hard nodig hebben.

Aangezien de meerderheid van de zwangere vrouwen in Nederland haar (vroege) zwangerschapscontroles bij een eerstelijns verloskundige heeft, is het van belang om te weten of vrouwen met een indicatie voor aspirine goed en tijdig worden bereikt. Door het beantwoorden van deze vragenlijst, helpt u ons om hier meer inzicht in te krijgen. U kunt geen goede of foute antwoorden geven. De vragenlijst is geen test over de kennis over aspirine in de zwangerschap. De vragenlijst is opgesteld door de arts-onderzoeker, klinisch verloskundige en gynaecologen uit het Amsterdam UMC locatie VUmc in nauwe samenwerking met de Hellp Stichting. Wij vragen u de vragenlijst naar eerlijkheid in te vullen om een waarheidsgetrouw beeld van de huidige praktijk te krijgen. Om u de vrijheid te geven om de vragenlijst eerlijk in te vullen, is de digitale vragenlijst geanonimiseerd. Wij kunnen dus niet achterhalen wie de vragenlijst heeft ingevuld.

Het invullen van de vragenlijst duurt ongeveer 20 minuten. Indien u wilt deelnemen, kunt u dat hieronder aanvinken. Uw toestemming zal hierdoor worden vastgelegd en de vragenlijst zal voor u toegankelijk worden.

Checkbox Hierbij bevestigd ik dat ik werkzaam ben als 1^{e} lijns verloskundige.
Checkbox Hierbij geef ik toestemming voor deelname aan de vragenlijst.

- ➔ *Indien beide checkbox waren aangevinkt, dan kwam de vragenlijst beschikbaar.*

Wij willen u nogmaals hartelijk bedanken voor uw tijd en deelname.

Translated version in English

Introduction

We would like to invite you to respond to our survey. First of all the purpose of the questionnaire is explained followed by what we hope to learn more about the implementation of aspirin in pregnancy among community midwives by means of your input.

The drug aspirin can be obtained through drugstores or pharmacies and has the brand name aspirin (generic name acetylsalicylic acid) or ascal (generic name carbasalate calcium) and is given during pregnancy to reduce the risk of uteroplacental complications such as hypertensive disorders in pregnancy and fetal growth restriction in women at increased risk.

An increasing number of pregnant women have an indication for aspirin use in pregnancy according to current guidelines. Up-to-date knowledge about placental complications in pregnancy and the risk-reducing effect of aspirin is established in the guideline 'Hypertensive disorders' of the Dutch Society

of Obstetrics and Gynaecology (NVOG) since December 2018 and in the module 'Acetylsalicylic acid' which was published in October 2019. The Royal DutchAssociation of Midwives (KNOV) published a module 'Ascal in case of elevated risk of hypertension' in January 2019 and a scientific response 'Ascal in women with a history of a dysmature newborn' in August 2020. These documents are supported by a KNOV leaflet for pregnant women on 'Aspirin'. This leaflet contains information for pregnant women with an indication for aspirin use in pregnancy. The information includes the reason for aspirin use during pregnancy and risk reduction, as well as practical information including timing of the gestational age to start aspirin use .

The purpose of this study is to gain a better understanding of:

- Different aspects around counselling aspirin use in pregnancy.

- Suggestions for further implementation of aspirin use in pregnant women with an indication.

We have calculated how many people need to complete the questionnaire to be able to give a reliable statement. This is approximately 330, based on approximately 2.500 practicing community midwives. With this, we reach the desired 95% confidence level and 5% has been taken into account as an acceptable margin of error. You understand that we need your input.

Since the majority of pregnant women in the Netherlands have their (early) pregnancy check-ups with a community midwife, it is important to know whether women with an indication for aspirin are reached and if reached, if this is in time. By answering this questionnaire, you will help us gain more insight into this subject. You cannot give right or wrong answers. The questionnaire is not a test of knowledge about aspirin in pregnancy. The questionnaire was prepared by the physician-researcher, clinical midwife and gynecologists from the Amsterdam UMC location VUmc in close cooperation with the Hellp Foundation. We ask you to fill out the questionnaire honestly to get a true picture of current practice. To give you the freedom to complete the questionnaire honestly, the digital questionnaire has been anonymized. Thus, we cannot find out who completed the questionnaire.

The questionnaire will take about 20 minutes to complete. If you wish to participate, please check below. Your consent will be recorded and the questionnaire will become accessible to you.

Checkbox I hereby confirm that I am working as a community midwife.
Checkbox I hereby give my consent to participate.

➔ *If both checkbox were checked, the questionnaire became available.*

Once again, we would like to thank you very much for your time and participation.

Supplementary Table 1: Overview of the questions of the survey

Background

Question	Answers	Nature of question
1. What is your age?		Open question
2. What is your gender?	a. Female<br>b. Male<br>c. Prefer not to answer	Multiple choice, one answer possible
3. In which year did you graduate as a midwife?		Open question
4. In which country did you graduate?	a. Netherlands<br>b. Belgium<br>c. Other<br>i. Please specify which country	Multiple choice, one answer possible
5. How many years do you work as a community midwife?		Open question
6. Besides your work as a community midwife, do/did you also work as a clinical midwife?	a. Yes<br>i. Please specify how many years<br>b. No	Multiple choice, one answer possible
7. In which province do you work?	a. Drenthe<br>b. Flevoland<br>c. Friesland<br>d. Gelderland<br>e. Groningen<br>f. Limburg<br>g. Noord-Brabant<br>h. Noord-Holland<br>i. Overijssel<br>j. Utrecht<br>k. Zeeland<br>l. Zuid-Holland	Multiple choice, multiple answers possible
8. Do you work in a city and/or village?	a. City<br>b. Village<br>c. Both	Multiple choice, one answer possible
9. How many midwives are working in your practice?		Open question
10. How many pregnant women are having the check-ups in your practice?		Open question

Advising about aspirin in pregnancy

Question	Answers	Nature of question
11. Are you familiar with the module of the Royal Dutch Association of Midwives about aspirin in pregnancy?	a. Yes<br>b. No<br>c. Don't know	Multiple choice, one answer possible
12. Are you familiar with the Royal Dutch Association of Midwives client folder about aspirin in pregnancy?	a. Yes → go to question 13<br>b. No → go to question 14<br>c. Don't know → go to question 14	Multiple choice, one answer possible

13. Do you hand out the client folder to women with a (possible) indication for aspirin?	a. Always<br>b. Often<br>c. Regularly<br>d. Sometimes<br>e. Never	Multiple choice, one answer possible
14. Did you ever set the indication for aspirin in pregnancy?	a. Yes → go to question 15<br>b. No → go to question 16<br>c. Don't know → go to question 16	Multiple choice, one answer possible

Prescribing aspirin

Question	Answers	Nature of question
15. Did you ever experience practical problems with prescribing aspirin?	a. Yes<br>b. No<br>If yes:<br>i. Please specify the problem<br>ii. In which percentage do you start to late with aspirin?<br>iii. In which percentage do you not start with aspirin?	Multiple choice, one answer possible<br><br>Open question
16. Via which route do you arrange a prescription of aspirin?	a. Consultation with a consultant obstetrician<br>b. Via obstetrician within obstetric multidisciplinary setting<br>c. General practitioner<br>d. Over-the-counter<br>e. Other<br>i. Please specify	Multiple choice, multiple answers possible
17. Which route for a prescription of aspirin do you prefer?	a. Consultation with a consultant obstetrician<br>b. Via obstetrician within obstetric multidisciplinary setting<br>c. General practitioner<br>d. Over-the-counter<br>e. Other<br>f. Please specify	Multiple choice, one answer possible
18. Do you advice women to take aspirin at night?	a. Yes<br>b. No<br>If no:<br>i. Do you give another or no advice? Please specify	Multiple choice, one answer possible
19. At which gestational age do you advice to start aspirin?	a. 8 – 12 weeks of gestation<br>b. 12 – 16 weeks of gestation<br>c. Other<br>i. Please specify	Multiple choice, one answer possible
20. At which gestational age do you advice to stop aspirin?	a. 34 weeks of gestation<br>b. 36 weeks of gestation<br>c. 38 weeks of gestation<br>d. Other	Multiple choice, one answer possible

i. Please specify

Possible indications for aspirin in pregnancy

21. Below is a list of clinical situations with possible indication for aspirin in pregnancy. Please answer if you would set an indication for aspirin in pregnancy or not.

Hypertensive disorders

Clinical situation	Answers	Nature of question
Pregnancy-induced hypertension with term birth in obstetric history	a. Yes, as isolated risk factor<br>b. Yes, if combined with other risk factor<br>c. No, no indication<br>d. No, not well informed	Multiple choice, one answer possible
PE/HELLP with term birth in obstetric history	a. Yes, as isolated risk factor<br>b. Yes, if combined with other risk factor<br>c. No, no indication<br>d. No, not well informed	Multiple choice, one answer possible
PE/HELLP with preterm birth between 34 – 37 weeks of gestation in obstetric history	a. Yes, as isolated risk factor<br>b. Yes, if combined with other risk factor<br>c. No, no indication<br>d. No, not well informed	Multiple choice, one answer possible
PE/HELLP with preterm birth <34 weeks of gestation in obstetric history	a. Yes, as isolated risk factor<br>b. Yes, if combined with other risk factor<br>c. No, no indication<br>d. No, not well informed	Multiple choice, one answer possible

Fetal growth restriction

Clinical situation	Answers	Nature of question
FGR with term birth in obstetric history	a. Yes, as isolated risk factor<br>b. Yes, if combined with other risk factor<br>c. No, no indication<br>d. No, not well informed	Multiple choice, one answer possible
FGR with preterm birth between 34 – 37 weeks of gestation in obstetric history	a. Yes, as isolated risk factor<br>b. Yes, if combined with other risk factor<br>c. No, no indication<br>d. No, not well informed	Multiple choice, one answer possible
FGR with preterm birth < 34 weeks of gestation in obstetric history	a. Yes, as isolated risk factor<br>b. Yes, if combined with other risk factor<br>c. No, no indication<br>d. No, not well informed	Multiple choice, one answer possible

Auto-immune diseases

Clinical situation	Answers	Nature of question
SLE	a. Yes, as isolated risk factor<br>b. Yes, if combined with other risk factor<br>c. No, no indication<br>d. No, not well informed	Multiple choice, one answer possible

APS	a. Yes, as isolated risk factor<br>b. Yes, if combined with other risk factor<br>c. No, no indication<br>d. No, not well informed	Multiple choice, one answer possible
**Maternal diseases**		
**Clinical situation**	**Answers**	**Nature of question**
Chronic hypertension	a. Yes, as isolated risk factor<br>b. Yes, if combined with other risk factor<br>c. No, no indication<br>d. No, not well informed	Multiple choice, one answer possible
Diabetes type I or II	a. Yes, as isolated risk factor<br>b. Yes, if combined with other risk factor<br>c. No, no indication<br>d. No, not well informed	Multiple choice, one answer possible
Chronic kidney disease	a. Yes, as isolated risk factor<br>b. Yes, if combined with other risk factor<br>c. No, no indication<br>d. No, not well informed	Multiple choice, one answer possible
**Other situations**		
**Clinical situation**	**Answers**	**Nature of question**
Nulliparity	a. Yes, as isolated risk factor<br>b. Yes, if combined with other risk factor<br>c. No, no indication<br>d. No, not well informed	Multiple choice, one answer possible
Advanced maternal age of ≥ 40 years	a. Yes, as isolated risk factor<br>b. Yes, if combined with other risk factor<br>c. No, no indication<br>d. No, not well informed	Multiple choice, one answer possible
Pregnancy interval ≥ 10 years	a. Yes, as isolated risk factor<br>b. Yes, if combined with other risk factor<br>c. No, no indication<br>d. No, not well informed	Multiple choice, one answer possible
Obesity with BMI ≥ 35 kg/m^2	a. Yes, as isolated risk factor<br>b. Yes, if combined with other risk factor<br>c. No, no indication<br>d. No, not well informed	Multiple choice, one answer possible
Family history of PE/HELLP	a. Yes, as isolated risk factor<br>b. Yes, if combined with other risk factor<br>c. No, no indication<br>d. No, not well informed	Multiple choice, one answer possible
Multiple pregnancy	a. Yes, as isolated risk factor<br>b. Yes, if combined with other risk factor<br>c. No, no indication<br>d. No, not well informed	Multiple choice, one answer possible

Egg cell donation	a. Yes, as isolated risk factor<br>b. Yes, if combined with other risk factor<br>c. No, no indication<br>d. No, not well informed	Multiple choice, one answer possible
Perinatal death caused by placental insufficiency in obstetric history	a. Yes, as isolated risk factor<br>b. Yes, if combined with other risk factor<br>c. No, no indication<br>d. No, not well informed	Multiple choice, one answer possible
Unexplained perinatal death in obstetric history	a. Yes, as isolated risk factor<br>b. Yes, if combined with other risk factor<br>c. No, no indication<br>d. No, not well informed	Multiple choice, one answer possible

*In case at least one of the above clinical situations was answered with 'No, not well informed', go to question 22. If not, go to question 23.

Suggestions

Do you advice aspirin in other clinical situations?	a. Yes<br>i. Please specify<br>b. No	Multiple choice, one answer possible

Clinical practice

Question	Answers	Nature of question
22. How would you like to be better informed about aspirin in pregnancy?	a. Extending the module of the Royal Dutch Association of Midwives<br>b. Written information of other resources<br>c. E-learnings<br>d. National website<br>e. Contact person per region for consultation (consultant obstetrician)<br>f. Other<br>i. Please specify	Multiple choice, multiple answers possible
23. Do you see your women in time to start aspirin in time?	a. Yes<br>b. No<br>c. Don't know	Multiple choice, one answer possible
24. Do you have enough time to counsel about aspirin?	a. Yes, almost always → go to question 26<br>b. Most of the time → go to question 26<br>c. Sometimes → go to question 25<br>d. Never, barely ever → go to question 25	Multiple choice, one answer possible
25. Do you have suggestions to help you to create enough time to counsel?		Open question
26. Do you check adherence?	a. Yes, almost always<br>b. Most of the time<br>c. Sometimes<br>d. No, barely ever	Multiple choice, one answer possible

27. In case of doubts about the safety of aspirin in pregnancy in you women, do you refer to The Netherlands Pharmacovigilance Centre Lareb (www.lareb.nl) as reliable source?	a. Yes<br>b. Sometimes<br>c. No	Multiple choice, one answer possible
28. Do you check side effects?	a. Yes, almost always<br>b. Most of the time<br>c. Sometimes<br>d. No, barely ever	Multiple choice, one answer possible
29. Should we generally inform all pregnant women about aspirin in pregnancy?	a. Yes<br>b. No<br>c. Don't know<br>Please specify	Multiple choice, one answer possible

PE, preeclampsia; HELLP, haemolysis elevated liver enzymes low platelets syndrome; FGR, fetal growth restriction; SLE, systemic lupus erythematosus; APS, antiphospholipid syndrome; BMI, body mass index.

© 2024 bij de Weg J.M. et al.
